# Evaluation of pilot community art-based workshops designed for Ukrainian refugee children

**DOI:** 10.3389/frcha.2023.1260189

**Published:** 2023-11-03

**Authors:** Steve Lukito, Michaela Wenkert, Inna Hryhorovych, Svitlana Opanasenko, Laura Timms, William Yule, Dennis Ougrin

**Affiliations:** ^1^ Department of Child and Adolescent Psychiatry, Institute of Psychiatry, Psychology and Neuroscience, King’s College London, London, United Kingdom; ^2^ Independent Researcher, London, United Kingdom; ^3^ St Mary’s Ukrainian School, London, United Kingdom; ^4^ Children and War UK, London, United Kingdom; ^5^ Department of Psychology, Institute of Psychiatry, Psychology and Neuroscience, King’s College London, London, United Kingdom; ^6^ Youth Resilience Research Unit, WHO Collaborating Centre for Mental Health Services Development, Centre for Psychiatry and Mental Health, Wolfson Institute of Population Health Queen Mary University of London, London, United Kingdom

**Keywords:** refugee children, community-art workshops, feasibility, acceptability, psychological wellbeing, service evaluation

## Abstract

**Objectives:**

Approximately 8 million Ukrainians have been displaced by the war in Ukraine and five million children had their education disrupted. Here, we report an evaluation of (1) the feasibility (i.e., recruitment), (2) the acceptability (i.e., attendance, participants' views) and (3) the influence of a pilot community art-based project on the well-being, health behaviour and socialisation of Ukrainian refugee children in London, UK.

**Methods:**

Twenty-two refugee children aged 4–14 years from St Mary's Ukrainian school in London took part in five weekly art workshop group sessions led by a team of volunteer independent artists based in a community art studio in West London in collaboration with Children and War UK. Analyses were conducted on measures of the children's psychological well-being, health behaviour, and socialisation; collected from participating children and their parents through the workshops.

**Results:**

The community art workshops received sufficient interest from parents during recruitment. Child participants and their parents expressed overwhelmingly positive views and high satisfaction towards the workshops and their activities. While the workshops were conducted without a control group, changes in psychological well-being and health behaviour and socialisation were in the expected direction. The workshops were associated with reduced impact of intrusive re-experiencing of traumatic events (*p* = .021), negative emotion (*p*s = .006–.043; rated by children and by their parents, respectively), and sleep disturbance (*p *= .015). Mood and motivational states increase relative to before activities within sessions (*p*s = .001–.023).

**Conclusions:**

The artist-led workshops are a valuable community project associated with high satisfaction and potentially increased well-being in Ukrainian refugee children. A provision for a larger number of participants should be considered.

## 1. Introduction

Approximately 8 million Ukrainians have been displaced and five million children had their education disrupted by the war in Ukraine. By March 2023, over 165k refugees from Ukraine were recorded in the UK (1). The refugees, mostly women and children as adult men are banned from leaving the country (2), needed to rapidly adjust to the local customs, speak a new language, and form social networks within the new community they are thrown into. Such stressors, on top of potential exposure to war violence, could lead to mental health difficulties among refugee children (3). Access to services that could help relieve some of these stresses may be useful to promote well-being in the children.

We describe here a community art-based project led by a team of volunteer artists in collaboration with health, education and academic professionals, which was formed in April 2022 as an immediate response to the needs of newly arriving displaced Ukrainian families in London. The project consisted of five community art workshops that were open to Ukrainian refugee children. Each workshop was dedicated to creating or decorating art objects using mixed-media techniques. Similar to other community art projects in the displaced communities (4–6), this project was aimed at promoting enjoyment, health, socialisation, and health behaviour (7–9). Furthermore, the project was aimed to enable skill learning, creativity, and to build a renewed sense of agency (10), facilitated by the community art principle that emphasizes participatory, creative “experience” and social processes rather than the final aesthetic of the produced artwork, which provides a non-elitist entry into the arts for all community members (11).

As well as being a direct response to the community needs, the workshops were created as a pilot for a larger community art project for Ukrainian refugee children and potentially other displaced communities in the future. To provide evidence-based support for future projects, we evaluated (a) the feasibility (i.e., recruitment, data collection), (b) the acceptability of the workshops (i.e., attendance, participants' views about the workshops) and (c) the influence of the workshops on the well-being, health behaviour and socialisation and the children's enjoyment using secondary data collected from the refugee children and their parents. We hoped to provide information about the feasibility of conducting similar future community art-based projects catered for the needs of Ukrainian refugee children, to demonstrate the positive influence, or the absence of negative effect, of the workshop sessions on the well-being of the participating children as reported by themselves and their parents, and to finally reflect on any lessons learned from the available data.

## 2. Methods

### 2.1. Design

This paper outlines secondary analyses of observational data gathered from the art-based community project to examine the feasibility, acceptability, and influence of the workshops on the well-being and healthy behaviour of the children (Local ethics approval No. LRS/DP-22/23-34303). These workshops were delivered to non-randomised groups of Ukrainian children without control participants.

### 2.2. Community art workshops

The community-art workshops, with a working title *My World Workshop*, took place from June to July 2022 and were organised by a team of volunteer artists in collaboration with the Children and War UK, founded and curated by the first author MW. These workshops were carried out in a community art studio in West London. The workshops involved a team of independent artists and volunteers comprising school educators from a London-based Ukrainian School, clinicians, and some higher education researchers.

The project consisted of five weekly, half-day workshop sessions, where children created or decorated art objects. These workshop sessions received input from different disciplines including art, health science, and pedagogy, and were critically informed by the *Displacement Art* movement (12) and object theory (13). The children's experience of displacement and exposure to war were not explicitly disclosed, but there was an anticipation that the art objects and their creation might reflect thoughts or insights that were related to experiences of geographical dislocation and forced migration (14) that might affect the children during the workshop. Therefore, each session was organised with Ukrainian-speaking trauma-specialist counsellors on site.

The sessions were curated with the recurring themes of “home”, and “hope”, selected in recognition of the children's displacement, while both honouring the memories of home and nurturing positive experiences and belonging in their new environment, in accordance with the community-art principle, displacement art, and object theory (12). As such, the sessions were built around art activities that combined mark-making and object-making that reflect on both themes. During the workshop sessions, children were presented with blank, symbolic *tabula rasa* objects, paper, clay, or fabric, to creatively mark-make upon, such that the objects brim with meaning. In addition to the individual work, some exercises included the making of communal art pieces to encourage the children to create their community and sense of collective agency.

**Figure 1:**
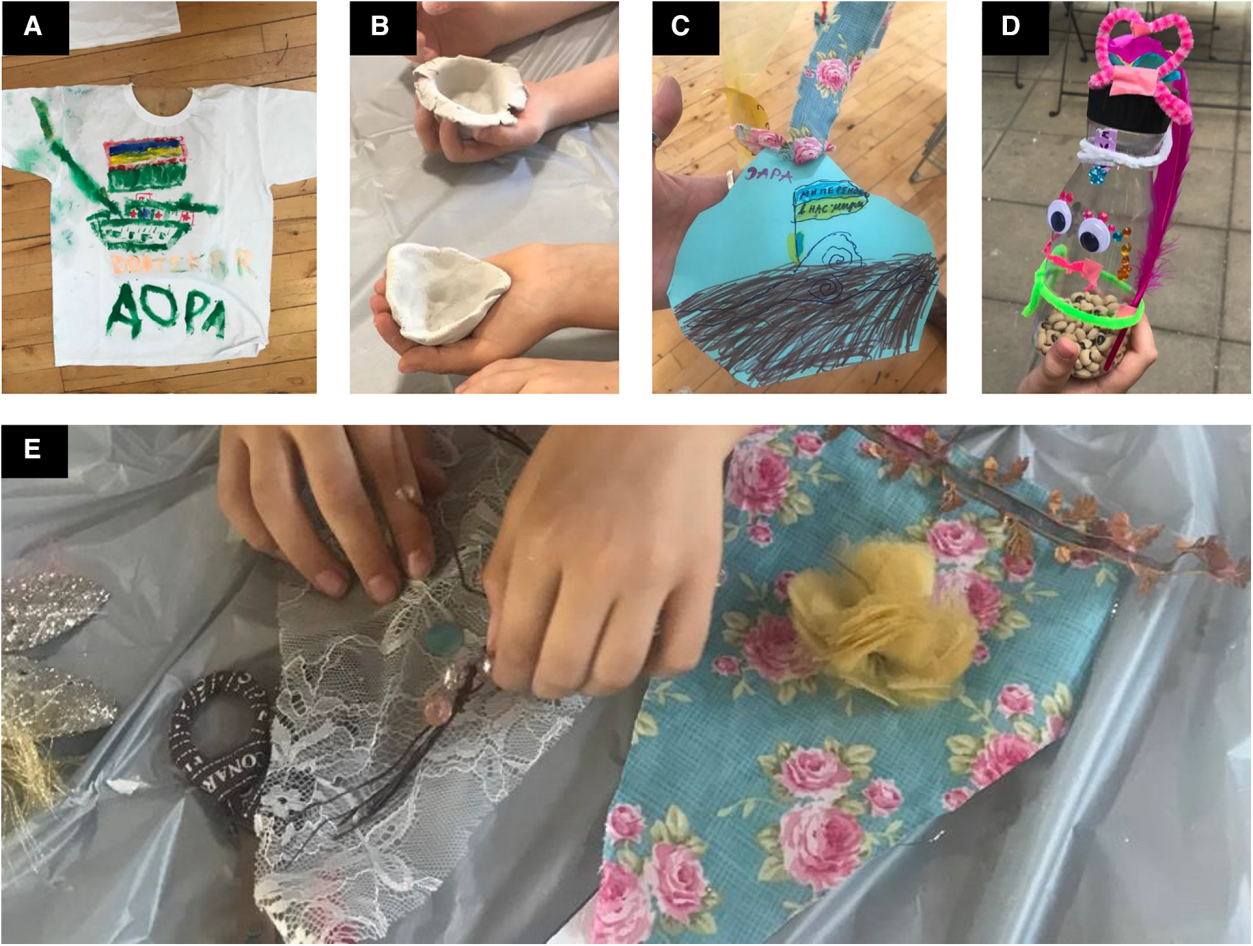
Example Art works produced by the children. Photographs of the art works produced by some children: (**A**) a t-shirt with a child's drawing of the war in Ukraine, (**B**) clay “hugging” bowls made of air-drying clay and a clay pinch-bowl technique, (**C**) a wish catcher with a message to hang onto the communal hoop with a strichka ribbon tagged with a drawing of a Ukrainian flag saying “We have won. We have peace”, (**D**) a percussion instrument made of a found material, in this case a plastic bottle, decorated with bows and stickers, and (**E**) a child places haberdashery, beading, and lace onto fabric bunting triangles. Photographs: Michaela Wenkert 2022©.

Each session was focused on one art project: (a) Workshop 1: *Decorating a Key* invited each child to ornately gild a lucky house key, and to draw/write a secret wish on a paper tag tied to the key; (b) Workshop 2: *Designing Own T-shirts* gave children the opportunity to self-express on a white t-shirt serving as a blank canvas; which was accompanied by a group activity *the Wish Catcher*, where the children created and tied personal paper messages on a giant, communal mobile hoop, in keeping with Ukrainian folklore traditions of hanging *strichka* (ribbon) that marks celebratory feasts, symbolising fortune; (c) Workshop 3: *Working with Clay* taught the children the skill of making “huggable” pinch bowls inspired by the Japanese Wabi-Sabi technique that celebrates the human marks of the maker, transience, and imperfections (15); (d) Workshop 4: *Make Your Own Bunting* enabled the children to create their own decorative style for their rooms at home, using collage, sewing, and fabrics; and finally (e) Workshop 5: *Percussion Music* taught the children to decorate percussion instruments made from white plastic bottles filled with dried pulses. The finished instruments were used in a free-style jam session, led by a professional musician (Figure 1).

### 2.3. Participants

Participants were female and male pupils attending the St Mary's Ukrainian School in London. The pupils were initially screened using the Child Revised Impact of Events Scale (CRIES-8) by teachers, and 24 pupils with higher scores were invited to take part in the community art workshops. The inclusion criterion was refugee children aged from 4 to 14 years. The participants' parents provided written informed consent for their children's data to be used as part of this publication.

### 2.4. Measures

A range of validated and non-validated questionnaires and inventories were used during the project. Some measures were adapted and shortened from validated measures to reduce the burden on the children. The measures assessed the mental health and psychological well-being, socialisation, and enjoyment of the children, as well as the feasibility of the families attending the workshops. The measures were translated when a Ukrainian version was unavailable. Unless otherwise stated, all questionnaires were completed up to one week before and immediately after the completion of the 5-week workshops.

#### 2.4.1. Child revised impact of events scale (CRIES-8)

The self-rated CRIES-8 was used to measure the impacts of traumatic events in the forms of intrusive re-experiencing of the events and avoidance of reminders/feelings associated with those events (16). This 8-item scale was designed for children aged 8 years and above who can read independently. It contains 4 items measuring intrusion and 4 items measuring avoidance. It yields a total maximum score of 40, with higher scores indicating higher post-traumatic stress symptoms.

#### 2.4.2. Emotional symptoms questionnaire

The Emotional Symptoms Questionnaire was adapted from the Adolescent Wellbeing Scale (17). The scale was chosen instead of the self-rated Strength and Difficulties Questionnaire below due to its accessible wordings for children. To reduce burden for the children, we used six items of the scale to explore their emotional symptoms: “I enjoy the things I do as much as I used to”, “I like talking to my friends and family”, “I feel like crying”, “I feel very bored”, “I look forward to things as much as I used to”, “I am easily cheered up”. The questionnaire yielded a maximum total score of 12, where higher scores indicate more negative emotions.

#### 2.4.3. Strength and difficulties questionnaire (SDQ)

The SDQ (18), is a 25-item screening questionnaire assessing five domains of emotional symptoms, conduct problems, hyperactivity/inattention (ADHD) symptoms, peer relationship problems and prosocial behaviour. Negative emotional symptoms of the children, reported by their parents, were indexed using emotional symptoms domain of the SDQ with a total score ranging from 0 to 10, where higher scores indicate more negative emotions.

#### 2.4.4. Columbia impairment scale (CIS)

The CIS (19) measured general impairment of different domains of functioning including relationships with family members and peers and academic performance at school. The parent-rated scale contained 12 items, and each was scored from 0 (No problem) to 4 (Very bad problem), leading to a maximum total score of 48, where higher scores indicated higher functional impairment.

#### 2.4.5. Sleep questionnaire

The brief non-validated sleep questionnaire was created based on the prompts question of the Kiddie Schedule for Affective Disorders and Schizophrenia (K-SADS) (20). The parent-rated questionnaire contains four items: “Does your child have problems falling asleep?”, “Does your child wake up at night?”, “How frequently does your child have nightmares?”, and “Is it difficult to wake your child up in the morning?”, each rated on a scale from 0 = “not at all”, 1 = “1–2 days”, 2 = “3–4 days” and 3 = “5 days or more”, yielding a total maximum score of 12, where higher scores reflect more sleep problems.

#### 2.4.6. Socialisation questionnaire

The brief 5-item non-validated Socialisation Questionnaire explored the workshop's role in encouraging and facilitating socialisation in the children. The inventory contained 5 items: “I like spending time with others in the workshops”, “The workshops help me make new friends”, “The workshops make me feel less lonely”, “I talk more often/with more people during the week”, and “I am confident in new situation”, rated 0 = “True”, 1 = “Somewhat True”, 2 = “Not True”. The total maximum score is 10, with higher scores indicating higher socialisation difficulties.

#### 2.4.7. Mood and motivation state questionnaire

The Mood and Motivation State Questionnaire was adapted from Maurizio et al. (21) and assessed the mood and motivational state of the children before and after activities in Sessions 2, 3 and 4. The self-rated questionnaire contained 4 items each consisting of opposite statements, from “Bored” to “Interested”, “Bad Mood” to “Good Mood”, “Tired” to “Awake”, and “Stressed” to “Not Stressed”, to be rated from 1 to 5, which will yield a total score up to 20, where higher scores indicate better mood or motivation.

### 2.5. Workshop planning and procedure

Prior planning of the workshops took place between April and June 2022 and consisted of meetings between MW, DO and representatives from the Ukrainian school and Children and War UK Charity, which helped formulate the workshops' objectives and required organisation. All members of the team received training on trauma provided by two qualified clinicians (DO, LT). The workshops were co-designed by artists, psychologists, and educational staff members, and underwent pre-piloting involving some Ukrainian families and the local community.

Each workshop started with a “welcome” and some refreshments for the children and their parents. Then the children were led to the art studio while their parents had the opportunity to socialise in an adjoining welcome room. Children were seated typically in small groups of 5–8 around workshop tables. They were introduced to the session and given a demonstration of the workshop activity in Ukrainian. Then they were given art supplies and encouraged to freely engage in creative expressions. Up to ten volunteers took part in each session to support the children's activities. Several were Ukrainian speaking and provided communication translations between the children and the English-speaking volunteers. Most activities were easily demonstrated visually. Each session was concluded with refreshments and children were encouraged to bring their artwork home with them. Data collection was conducted by Ukrainian-speaking volunteers under the supervision of the first author SL.

### 2.6. Analysis plan

The influence of the workshops on trauma, mood, daily functioning, and sleep was explored by comparing these dependent variables before and after the workshop completion using a *linear mixed model*, with time as fixed effects, and compound symmetry covariance structure. *Post-hoc* explorations adding (1) age group (i.e., preschool [aged <5 years] and school age [≥5 years and over]) and time × age group or (2) sex and time × sex to investigate whether age or sex moderate the pre- to post-workshop changes, i.e., whether the main interaction effects of time × age or time × sex as indicated by *F*-statistics, were significant. As there were only few pre-school children with substantial missing data, we also carried out a sensitivity analyses including only school age children. Furthermore, the influence of each session on the dependent variable mood and motivational state of the participants was examined by comparing their ratings before and after each session using paired *t*-tests.

All the children's experience of friendship and their and their parents' views about the workshops were assessed by combining the percentage ratings of “somewhat” and “yes” on each item denoting their presence. Free-text answers from the families were translated into English from Ukrainian and grouped according to themes and described narratively. All quantitative analyses were conducted with IBM SPSS Statistics for Windows 27 (Armonk, NY).

## 3. Results

### 3.1. Recruitment and data collection

Fifty-three pupils were screened using the CRIES-8 by teachers, and 24 pupils with the higher scores were invited to take part in the community art workshops. Of the 24 pupils invited, eight declined to participate. These spaces were allocated to other pupils on a first-come first-serve basis, resulting in a total of twenty-two participants in the workshops (9 females and 13 males) aged from 4 to 13 years (mean = 7.36; standard deviation = 2.53 years). The children and their parents fully completed the questionnaires on site. However, there was no mechanism to gather data from the families outside the sessions. Therefore, datasets were completely missing from the families when they missed a session.

### 3.2. Acceptability of the workshops

#### 3.2.1. Workshop attendance

The families attended on average 3.1 of 5 sessions on average (median = 3, range 1–5, *n* = 23), with 60.8% of the participants attending at least three out of 5 sessions. Sessions 1–5 were attended by 14, 15, 16, 13 and 15 children, respectively. Data from *n* = 15 parents indicated that the children missed sessions primarily due to sickness (*n* = 4) and travelling (*n* = 2). Anecdotal information was provided by school staff who stated that a few families missed some sessions due to parents' attendance at peace protests against the war.

#### 3.2.2. Children's and parents' views about the workshops

**Figure 2:**
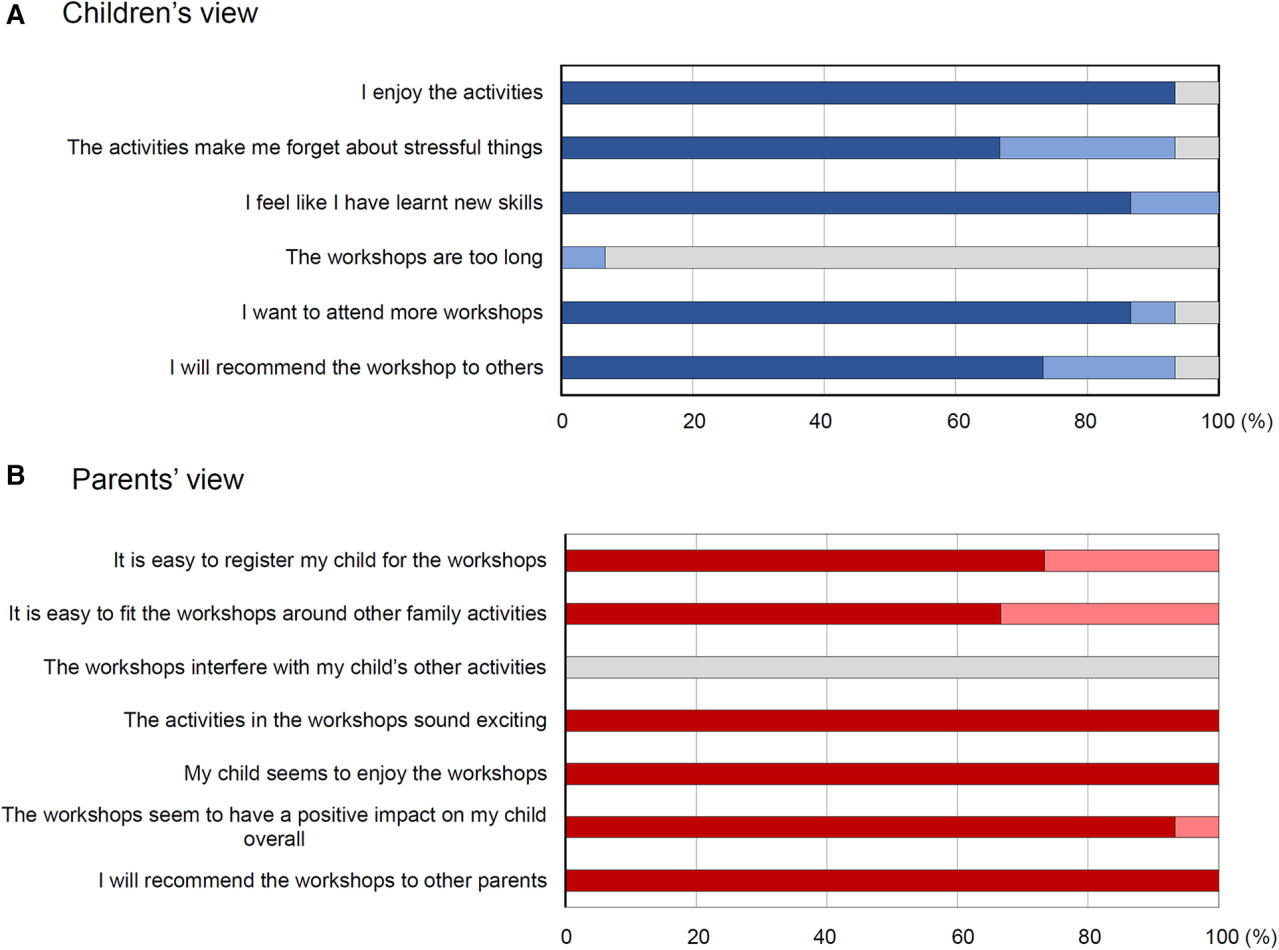
Children's and Parents’ views about *My World Workshops*. Children's (**A**, blue) and parents’ (**B**, red/pink) views on the workshops and how they can be further improved. Darker blue/red indicates “true”, lighter blue/pink indicates “somewhat” and grey indicates “false”.

Most children who provided data at the end of the workshops (*n* = 15) reported that they enjoyed the activities (93.3%), and that the activities helped them forget about stressful things (93.3%). They felt that they have learnt new skills (100%). Only one child reported that the workshops were somewhat too long (6.7%). Most wanted to attend more workshops (93.3%) and would recommend the workshops to others (93.3%) (Figure 2A). Free-text feedback was given by ten child participants, mostly related to the workshops' contents (*n* = 3) and the desire to have more sessions (*n* = 3) (see Table 1 for themes and quotes). Lastly, the three most popular workshops among the children were: (1) “Designing Own T-shirts”, (2) “Percussion Music”, and (3) “Working with Clay”.

**Table 1:** Participants views on how to further improve the workshops.

Themes	*n*	Quotes
Children's views (*n* = 10)		
Workshop content suggestion	3	“To have more stories and ideas”, “entertainment for older teenagers”, “to have more supplementary materials”.
More sessions	3	“To learn more”, “continue”, “the first one, too little”.
Satisfaction/gratitude	2	“I am happy with everything”, “thank you”
Not knowing what to improve/no improvement needed	2	“I do not know”, “nothing”
Parents’ views (*n *= 10)		
Workshop content suggestion	1	“Go out to the yard/open space’
More sessions	2	“We’d like to attend more—It's not easy to say goodbye”, “continue”
Satisfaction/gratitude	6	“I was pleased with everything”, “all super”, “I enjoyed it all—very interesting session”, “all super, thanks”, “all is good, thank you”, “all is well”
No improvement needed	1	“Nothing was needed everything was fine”.

Themes arising from the free text answers from the participants and their families.

All parents providing data at the end of the workshops (*n* = 15) found it easy to register their children into the workshop (100%), and to fit in the workshops around other family activities (100%). The workshops did not interfere with the children's other commitments (0%). Parents thought that the activities were exciting (100%) and that their children appeared to enjoy and to derive positive influence from the workshops (100%). They would recommend the workshops to other parents (100%) (Figure 2B). Parents' free-text suggestions (*n* = 10) revolved around similar themes as their children, with the theme of satisfaction and/or gratitude being the most dominant (*n* = 6), followed by the desire to have more sessions (*n* = 2) (Table 1).

### 3.3. The workshops' influence on the children's well-being and socialisation

**Figure 3:**
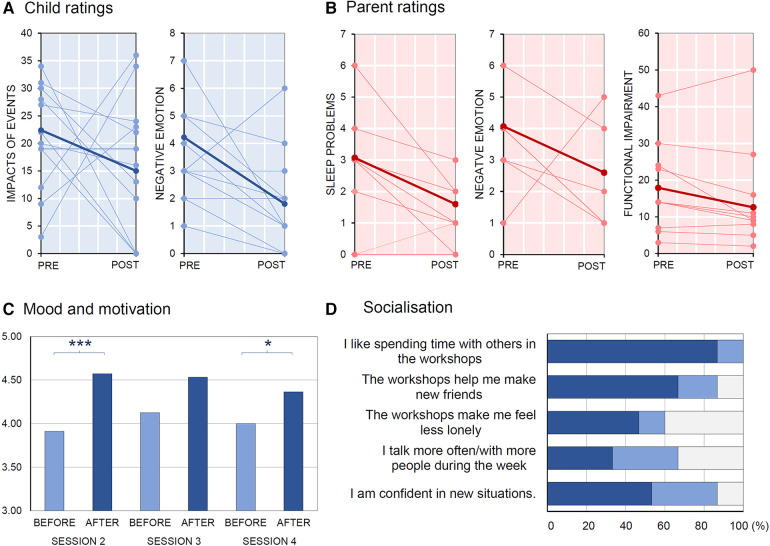
Psychological well-being and socialisation of the participants. Reports of (**A**) child-rated impacts of traumatic events and negative emotions, (**B**) of parent-rated sleep problems, negative emotions, and functional impairments measured pre- and post-workshops, (**C**) mood and motivational state before and after a session, and (**D**) socialisation of the children post-workshops. Child-rated measures are coloured blue, parent-rated measures about the children are coloured pink. Darker blue/red indicates “true”, lighter blue/pink indicates “somewhat” and grey indicates “false”.

Pre- and post-workshop ratings are presented in Table 2. Children self-reported impact of traumatic events score appeared lower at post- relative to pre-workshop period, although non-significantly, *t*(21.1) = 2.04, *p* = .054. This was driven by lower intrusive re-experiencing of traumatic events, *t*(20.1) = 2.51, *p* = .021 to the children post-workshops, but not by avoidance of reminders/feelings associated with the event, which did not differ significantly between pre- and post-workshops, *t*(20.8) = 1.30, *p* = .21. The children also reported significantly reduced negative emotion, *t*(10.1) = 3.50, *p* = .006. Parents reported the children to have significantly reduced negative emotion, *t*(16.2) = 2.19, *p* = .043, and sleep disturbance, *t*(9.69) = 2.96, *p* = .015. Finally, they also reported a weak but non-significant reduction of functional difficulties from pre- to post-workshops, *t*(10.4) = 1.999, *p* = .073 (Figures 3A,B).

**Table 2:** Pre- and post-workshop scores.

Measures	Pre M (SD)	Post M (SD)
Child report		
Impacts of events	22.4 (8.90)	15.0 (11.6)
Avoidance	11.7 (5.99)	8.80 (6.84)
Intrusion	10.7 (4.42)	6.20 (5.52)
Negative emotion	4.22 (2.26)	1.80 (1.61)
Parent report		
Negative emotion	4.07 (1.90)	2.60 (1.64)
Functional difficulties	17.9 (11.9)	12.6 (12.2)
Sleep problems	3.07 (2.09)	1.60 (1.72)

Child report of negative emotions were measured using the adapted Adolescent Wellbeing Scale, while the Parent report of their children's negative emotions were measured using the score of emotional symptoms of the Strength and Difficulties Questionnaire.

*Post-hoc* analyses revealed no significant interaction between age group or sex and the pre- and post-workshop changes of the dependent variables, *F*_age × time_ (*df* = 16.6–25.6) ≤ 1.32; *p*s_age × time_ = .39–.95, and *F*_sex × time_ (*df* = 8.65–18.9) ≤ 1.31; *p*s_sex × time_ = .28–.70. Sensitivity analyses involving only school-age children revealed a pattern of diminishing workshop influence which remained significant only in the context of self-reported negative emotion, *t*(7.73) = 3.09, *p* = .015, and parent-reported sleep disturbance, *t*(8.81) = 2.80, *p* = .021, and non-significant for all other measures, i.e., parent-reported reduction of the children's negative emotion, *t*(12.7) = 1.99, *p* = .068 and functional difficulties from pre- to post-workshops, *t*(9.90) = 1.96, *p* = .079, and self-reported of trauma, *t*(18.0) = 1.62, *p* = .12, and intrusive trauma re-experiencing, *t*(16.6) = 1.99, *p* = .063, and avoidance, *t*(17.8) = 1.01, *p* = .33.

Children reported their mood and motivation scores increasing after compared to before the activities in Session 2 (*n* = 15), *t*(13) = 4.01, *p* = .001, and Session 4 (*n* = 13), *t*(12) = 2.60, *p* = .023. Their scores did not increase significantly in Session 3 (*n* = 16), *t*(15) = 1.97, *p* = .068 (Figure 3C) although the change of mean scores was in the expected direction.

Post-workshops, children reported that they liked spending time with others in the workshops (100%), that the workshops helped them make new friends (87%), feel less lonely (60%), talk more often/with more people during the week (67%), and feel more confident in new situations (87%) (see Figure 3D).

## 4. Discussion

This study aimed to provide an evaluation of (1) the feasibility, (2) the acceptability, and (3) the influence of the pilot community-art project *My World Workshops* on the psychological well-being, health behaviour and socialisation of the Ukrainian refugee children and their families, using secondary data analyses collected before, during and after the workshops.

The workshops showed an overall good level of feasibility for recruitment and data collection. There was a sufficiently high level of interest from the families to take part in the workshops, such that nearly all workshop spaces (95.8%) were taken even though some potential participants declined to take part during the initial invitation.

Completion of the questionnaires could pose a significant burden to children, but this appeared not to be the case during the data collection for the workshops. Therefore, it was promising that all participants, including the younger children, could complete all the questionnaires fully. The success could be attributed to the selection of shorter measures and the administration of the questionnaires by Ukrainian-speaking volunteers on sites. However, the absence of data collection mechanism outside sessions did lead to some missing data. Gathering data outside sessions is essential and is recommended for future workshops. This is likely to be a joint effort between school staff and Ukrainian-speaking volunteers/researchers.

The workshops showed an adequate level of acceptability from the point of view of attendance, with over 60% of participants attending at least three of the five sessions. More importantly, the families reported overwhelmingly positive views about the workshops. Most children derived enjoyment from the workshop activities, which helped alleviate stresses and allowed them to learn new skills as was the case in previous qualitative reports in displaced communities (4, 5). Most of the children expressed wishes to have further workshops and would recommend them to others. Parents indicated that it was not difficult to register their children into the workshops and to fit them in around other family activities. All parents viewed the workshops as exciting, enjoyable, and benefitting their children, and would recommend the community art workshops to others.

Our findings regarding the influence of the community art-based workshops on the children's psychological well-being, health behaviour and socialisation are generally positive, in line with past findings of the use of creative arts to increase well-being in young people [as reviewed by (22, 23)]. The art form used in the studies included in these reviews were typically performative, presumably chosen due to their appeal to older adolescents aged 11–18 years for whom the interventions were designed. This resonates with the free-text suggestion to include “entertainment for older teenagers” from one workshop participant. Should these workshops be made available for older adolescents, expanding the art medium of creative expressions to suit the wider age range might be beneficial.

The brief community-art workshops differed from the more intensive and/or longer-term art therapy (i.e., 12–15 sessions) provided for traumatised child refugees of a similar age range (24, 25). Nevertheless, these workshops appear promising for providing relief from trauma, and improving mood and sleep disturbances among child refugees with elevated trauma scores. Each workshop session, particularly in Sessions 2 and 4, is also associated with improved mood and motivational states after relative to before activities, indicating the rapid influence of the workshops on the children's emotional well-being. Taken together, these findings suggest that the community-art based workshops will find applicability in children who do not otherwise meet the threshold for receiving clinical care, either to influence their well-being in the longer term or to provide short-term emotional relief.

The influence of these workshops on the well-being of the children appeared not moderated by sex or age groups (preschool- vs. school-age), suggesting the versatility of the approach to different child subpopulations. When the analysis was repeated to include only school-age children, who constitute the largest portion of the sample, our findings suggest a downward trend in the influence of these workshops on children's well-being. However, it's important to note that such a trend might be a result of reduced statistical power for detecting effects in smaller subpopulations. Replicating the analyses with a larger sample that includes a more even distribution of preschool and school-age children is necessary to arrive at a more robust conclusion.

Finally, most children reported more socialisation and confidence after the completion of the workshops. Overall, the workshop completion thus appears to be associated with increased skill learning, creativity, and a renewed sense of agency, which fulfilled the core aim of the project (10).

There are some limiting factors to the interpretation of these findings. First, the absence of mechanisms for collecting data outside sessions from the family have led to missing data from participants who were absent from some sessions. The problem of missing data was partly mitigated by the linear mixed modelling statistical approach, but missing data could be associated with self-selection, which biases the conclusion of this evaluation in a positive direction. Second, our evaluation was based on observation from a small sample of participants, which limited the power for detecting smaller effects including potentially any moderating influences of subpopulation characteristics on the pre- to post-workshop changes. Third, while the association between the completion of these community-art-based workshops and the children's psychological well-being and health behaviour is promising, such potential benefits might still be attributable to non-specific developmental changes over time due to the absence of a control group. Future studies thus may consider the inclusion of an appropriate control group to establish more rigorous evidence for the specific benefits of these workshops for the children.

## 5. Conclusion

In conclusion, based on the available data, community art-based workshops appear to be feasible and acceptable, and they exert a positive influence on the psychological well-being, sleep behaviour and socialisation of Ukrainian refugee children that do not seem to be moderated by sex or age groups of the participants. The artist-led community-art workshops are valuable for Ukrainian refugee children. A provision for a larger number of participants should be considered, with an appropriate experimental control to yield rigorous evidence of the workshops' positive influence on the well-being of the refugee children.

## Data Availability

The data are available from the first authors upon reasonable request. Requests to access these datasets should be directed to steve.lukito@kcl.ac.uk.

## Author’s note

MW is an Independent Artist and Researcher in London, United Kingdom. LT is a clinical psychologist and trustee at Children and War UK, London, United Kingdom, and WY is Emeritus Professor of Applied Child Psychology at the Institute of Psychiatry, Psychology and Neuroscience, King’s College London, United Kingdom, and trustee at Children and War UK, London, United Kingdom.
